# Notes and News

**Published:** 1899-01-21

**Authors:** 


### NOTES AND NEWS.

(COLLECTED AND COMMUNICATED.)

HOSPITAL MEETING.

General Lying-in Hospital, York Road, Lambeth.—Annual meeting, half-past four p.m., on Wednesday, January 25th.

THE plague has appeared in Calcutta, though not to any great extent. At Madras and throughout the Mysore district a steady decline is reported.

AN arrangement to hold Sunday concerts in Aberdeen for the benefit of the Hospital Saturday Fund of that city has caused a disturbance, and the scheme has had to be abandoned.

THE death is announced of the Emeritus Professor of Physiology and Anatomy at Brussels, Dr. Gottlieb Gluge. His works on influenza and his great "Atlas of Pathological Anatomy" are well known.

THE recent gales are responsible for the partial destruction of a temporary isolation hospital at Bromsgrove, near Birmingham. Eight scarlet fever cases were in one of the tents which was completely blown away. The nurses were able to move the patients to another structure without injury.

THROUGH the generosity of the Right Hon. W. J. Pirrie, and with the consent of the White Star line, the launch of the "Oceanic" was made a means of benefiting the Royal Hospital at Belfast. One thousand seats on a wharf built by Mr. Pirrie were placed at the disposal of the committee of the Royal Hospital, and the price of the seats was arranged by them at 10s. each.

AMONGST the latest contributions received by the Prince of Wales's Hospital Fund for this year are the following annual subscriptions: Mr. W. W. Astor, £1,000; Clothworkers' Company, £1,000; Messrs. C. J. Hambro and Sons, £250; Messrs. Wernher, Beit, and Co., £250; the Duchess of Cleveland, £100; Sir Frederick Wills, £100; Mr. Edward Hore, £100; Mr. T. Spencer, £100; Messrs. J. Swire and Sons, £100; Mr. Henry L. Raphael, £100.

THE new cottage hospital at Chippenham has been opened by Lady Poynder, and is to serve as a Jubilee memorial.

IT is good news for dwellers in London that the Public Health Committee of the London County Council have offered a prize for the best design for a dust-cart, with a view to minimising the present extremely unpleasant tendency of these vehicles to shed a portion of their contents on windy days into the faces of pedestrians.

IN respect to the Barker Anatomical Prizes of the Royal College of Surgeons in Ireland a prize of £21 is offered for competition, and is open to any student whose name is on the Anatomical Class List of any school in the United Kingdom. The preparations entered must be placed in charge of the Curator before June 1st, 1899. The prize is offered for dissections to illustrate the anatomy of the larynx. The conditions under which the competition is to be carried out can be ascertained by applying to Curator of the Museums, J. Alfred Scott, M.D., F.R.C.S.I.

AT the National Hospital for the Paralysed and Epileptic, Queen Square, the following clinical lectures, free to gentlemen who are practitioners or students, will be held at half-past three on the following Tuesdays: January 24th, Dr. Ferrier, F.R.S., "Paraplegia"; January 31st, Dr. Ormerod, "Ataxy"; February 7th, Dr. Beevor, "On the Actions of Muscles"; February 14th, Dr. Tooth, "Cranial Nerves" (lantern demonstration); February 21st, Mr. Horsley, F.R.S., "Surgery of the Nervous System" (lantern demonstration); February 28th, Mr. Gunn, "The Value of the Visual Fields in Diagnosis"; March 7th, Mr. Ballance, "Surgery of the Nervous System"; March 14th, Dr. Taylor, "Syphilitic Diseases of the Nervous System"; and March 21st, Dr. Risien Russell, "Cerebral Tumours" (lantern demonstration).

WE publish the following copy of a placard which was circulated in Lucknow, and was printed in the *Indian Medical Gazette*: "Dr. M. S. Varis, M.B., C.M. Edin., consulting physician and surgeon. Consultation, all hours free, 9—11 A.M. Share Darvoza. Notice.—Dr. Sayad Mahomed Varis, surgeon. 'Good news to thee, O heart; a Jesus-like man has come.' Be it known to the seekers after bodily health and to those in the clutches of deadly diseases that the Aristotle of the times and Galen of the universe, Dr. Sayad Mahomed Varis, M.B., C.M., after learning the art of medicine and practising it in Great Britain, has come to this town (Lucknow). He studied for six or seven years in modern Athens, viz., Edinburgh, which is the capital of Scotland, and he obtained the diploma of a physician and surgeon; and there for three years he established himself in practice and performed Christ-like miracles. It is our good fortune that he has established himself here. It is hoped that whosoever will apply to him for treatment will fill his pocket with pearls of health. He lives close to Kaisar Bag, near Share Darvoza, opposite the telegraph office, in house No. 1. Patients can consult him all day."

